# *In vivo* and *in vitro* susceptibility and inflammatory response of postnatal mouse cortical neurons and glial cells to zika virus infection

**DOI:** 10.1371/journal.pone.0339900

**Published:** 2025-12-31

**Authors:** María-Angélica Calderón-Peláez, Myriam L. Velandia-Romero, Jaime E. Castellanos

**Affiliations:** Virology Group, Vice-Chancellor of Research, Universidad El Bosque, Bogotá, Colombia; Instituto Nacional de Salud Publica Centro de Investigaciones sobre Enfermedades Infecciosas, MEXICO

## Abstract

Zika virus (ZIKV) poses a significant threat to neural tissue, causing substantial damage to unborn children exposed to the virus *in utero*, with consequences that can manifest even after birth, despite being born with a normal head circumference. Regardless of the extensive research, the interactions between ZIKV and the nervous system cells remain insufficiently understood, particularly regarding how neuronal responses influence broader inflammatory and viral dynamics especially in postnatal stages of development. This study evaluated the susceptibility to ZIKV infection, viral replication, immune response, and survival of neurons, astrocytes and microglial cells during postnatal developmental stages, using both *in vivo* and *in vitro* mice models. *In vivo*, a non-lethal but extensive infection of neurons and microglia was shown. The infection caused a robust but controlled immune response with elevated levels of MCP-1, TNF-α, and IL-6, that prevented severe neuronal damage. *In vitro*, neurons exhibited high susceptibility to ZIKV, with elevated levels of pro-inflammatory cytokines and IFN-β, indicating a strong inflammatory response. In contrast, astrocytes and microglia displayed varied responses, contributing to a pro-inflammatory feedback loop. These findings offer critical insights into the cellular dynamics of ZIKV infection, enhancing our understanding of its effects during postnatal nervous system development. By clarifying the interactions between ZIKV and neuronal cell types, this study deepens the comprehension of the virus’s pathophysiology and its broader implications for neurodevelopmental outcomes, extending beyond the well-documented association with microcephaly.

## Introduction

The neuropathogenesis caused by the Zika virus (ZIKV) remains a topic of great interest. Discovered in 1947 [1] the virus remained relatively silent for decades. However, as it moved from Africa to Asia and then to the Americas [2], it mutated, leading to severe consequences for infected individuals, including Guillain-Barré syndrome (transient or chronic) in adolescents and adults [3], and congenital ZIKV syndrome (CZS) with effects like microcephaly [4,5], which is undoubtedly the most serious and catastrophic condition for children exposed to the virus *in utero* [6].

During the outbreak of 2015 and 2016, several models were established to explain various aspects of ZIKV infection and the development of neurological alterations [7]. Initially, the primary interest was to evaluate and confirm the neurotropic and neuropathogenic potential of the virus, as well as its ability to induce damage leading to microcephaly. Using 2D [8] or 3D cultures with different types of human or murine primary or cell lines, researchers demonstrated the pan-tropism of the virus and its infection of nervous tissue cells at early stages of development [9].

*In vivo* models were also employed, involving mice of different strains (immunocompetent, immunocompromised, or knockout) and individuals of other species (non-human primates, swine, etc.) inoculated via various routes [10]. These models used viruses isolated from different genotypes (African MR766, Asian, Asian-American) [11] and confirmed the vertical transmission of the virus, its tropism, infection, and high toxicity in progenitor cells of nervous tissue [12]. Additionally, these studies demonstrated that the strain of ZIKV that spread from Brazil to other American countries is more neurotropic, neurovirulent, and neuropathogenic compared to its Asian ancestor [13].

Although microcephaly is the most prominent feature of CZS, it is only present in some cases. In others, children develop secondary microcephaly or ocular anomalies during growth −30–36 months later- [14], hearing impairment [15] cerebral palsy, motor impairment, functional changes, and significant delays in neurocognitive development, even if born with a normal head circumference [16]. Neurodevelopmental delays have been identified in at least 30–35% of normocephalic ZIKV-exposed infants by 18 months of age in Central and South American countries, with most reports originating from Brazil and Colombia [16,17]. These delays most commonly affect the language and communication domains [18–20], significantly impacting the quality of life of children and their families [21]. Despite these findings, the factors promoting these outcomes and the cellular and molecular mechanisms associated with non-microcephalic CZS remain poorly understood [22,23].

Suckling mice inoculated between one and three postnatal days have also been used to evaluate the effect of ZIKV on the nervous tissue during these stages of murine development, which are comparable to intrauterine neurodevelopment in the third gestational trimester in humans [24,25]. These models have been instrumental for describing the immune response established in the tissue during infection. However, as with other neuroinfection models involving flaviviruses, this response is heterogeneous and depends on the model and virus used. For instance, some authors report the expression of molecules such as IFN I, II, and III by infected astrocytes [26], while other reports do not detect these molecules in the same cells [27] Infected or non-infected cells (neurons or glia) secrete pro- or anti-inflammatory molecules, which induce the activation of astrocytes, microglia, and endothelium. This, in turn, promotes the infiltration of immune cells due to alterations in the permeability of the blood-brain barrier (BBB), potentially increasing tissue damage, evading the immune response [28], or controlling the infection, as reported in some *in vivo* models [29].

When independently evaluating the effects of the virus using different models, it is challenging to discriminate the type of response each cell type exhibits to the virus. Therefore, using one-day-old immunocompetent neonatal mice (Balb/c), we evaluated *in vivo* and *in vitro* the susceptibility, survival, and immune response of cortical neurons, astrocytes, and microglial cells against a strain of ZIKV that circulated in Colombia during 2016, the country with the second highest number of Zika fever and CZS cases in South America [29].

## Materials and methods

### Animals and welfare considerations

All the protocols described below were endorsed by the Ethics Committee of the Universidad El Bosque (Bogotá, Colombia, record No. 013–2019) and the National Institute of Health of Colombia (record No. 11–2019), adhering to Colombian regulations of the international and Colombian standards for animal management.

#### Housing and husbandry.

The animals were housed in the animal facility at Universidad Nacional de Colombia, in an isolated room meeting all the necessary requirements for proper animal care. Adult male and female mice, along with their pups, were kept in appropriately sized ventilated cages (500 cm^2^ of floor space, model NexGen500, Allentown), covered with sterile wood chip (Aspen Chip and Lab Grande Aspen, NEPCO). The top of the cage held an external plastic 250 mL water bottle and a Whatman filter that allowed clean air exchange and protected the food (placed in a half pocket wire bar lid) with all the nutritional requirements for mice’s survival and reproduction. The cage had an enrichment (60 mm x 78 mm) for mice entertainment. All home cages were properly labeled, and the birth dates and the number of offspring at each birth were recorded. During the experimental period, the animals moved to clean cages with new food and water once a week.

#### Animal physical condition monitoring.

Every day during the experimentation period, we observed and recorded the typical behaviors of the animals, including maintenance, exploration, affiliate interactions, and sexual and maternal behaviors. Any abnormal behavior was promptly reported to the veterinarian.

#### Protocol for the early euthanasia/humane endpoints for animals who became severely ill/moribund during the experiments.

- **The specific criteria used to determine when adult animals should be euthanized:**
- **Adult animals:** In our model, adult mice were used exclusively as breeding pairs to produce the experimental pups. However, their body weight, body condition, and general behavior were monitored daily. Humane endpoints were applied based on the severity and persistence of clinical signs. Animals showing mild to moderate signs defined as ≤ 15% loss of body weight relative to baseline, a body condition score of ≤ 2/5 (prominent bones, reduced muscle mass), a hunched posture with marked lethargy and reluctance to move or explore, persistent piloerection and dull fur coat, were monitored closely for up to 48 h. If no improvement was observed or if clinical condition worsened, the attending veterinarian was notified, and humane euthanasia was performed. Adult animals showing severe signs were euthanized immediately. Such signs included:
  ◦Evidence of acute or chronic pain (e.g., vocalization, excessive grooming, or self-mutilation)
  ◦Palpable tumors
  ◦Respiratory distress (shallow breathing or frequent gasping)
  ◦Severe dehydration (skin remains tented after pinch test, tacky mucous membranes)
  ◦Neurological abnormalities (severe ataxia, tremors, or seizures)
- Importantly, none of the adult animals developed severe clinical signs during the experiments.
- **Neonatal Animals (Postnatal days 1–10):** Weight gain, thermoregulation, nursing behavior, and general responsiveness were monitored daily. Humane endpoints were defined according to the severity and duration of clinical signs. Pups showing mild to moderate signs: failure to gain weight, mild hypothermia, or slightly reduced suckling activity, were closely monitored for up to 48 h. If persistent weight loss or inability to gain weight was observed during this period, or if the clinical condition deteriorated, the attending veterinarian was notified, and humane euthanasia was performed. Immediate euthanasia was carried out if any severe signs were observed, including:
  ◦Continuous weight loss for 48 hours or persistent failure to nurse (inability to maintain latch on the dam’s nipple).
  ◦Extreme lethargy (absence of movement, minimal response to gentle tactile stimulation).
  ◦Continuous high-pitched vocalizations indicating unrelieved distress.
  ◦Obvious signs of severe dehydration (skin remains tented, dry mucous membranes).
  ◦Malformations or injuries incompatible with survival
- In such cases, the veterinarian was informed immediately, and euthanasia was performed by anesthetic overdose in accordance with institutional animal care and use guidelines.
- **Once animals reached endpoint criteria, the amount of time elapsed before euthanasia:** 1–2 h maximum
- **None of the experimental animals died before meeting the criteria for euthanasia.** However, two of the parental animals (that were only used to produce the pups for the experiments but were not used directly in the experiments) presented abnormal behavior. The veterinarian was immediately informed, and the animals were euthanized.

#### Euthanasia method for pups utilized in this research.

Postnatal pups were euthanized with an overdose of anesthetic (ketamine and xylazine).

#### Other relevant information.

- The duration of the experiment was 10 days for the *in vivo* model. For the *in vitro* model pups were euthanized at 1-day-old.
- The numbers of animals used: For *in vivo* model 12 animals per experiment (in two independent experiments), were infected at 1 day-old and kept until the 10-day post-infection. *In vitro* cultures were established with cells from 1-day-old pups as follows:
- Astrocytes and microglia: 1 pup per T-75-culture flask. These cells were purified and allowed to replicate. Because of this, a total of 8 animals were used for all the mentioned experiments.
- Neurons: 3 pups per experiment. Unfortunately, neurons are not replicative, so a total of 20 pups were used for all the mentioned experiments.
- How frequently animal health and behavior were monitored: Daily
- All researchers are trained in the use of animal models for scientific research. The first author (MACP) and the corresponding author (MLVR) oversaw the animals during the whole experimental process. Both are certified by The Colombian Association for the Science and Welfare of the Laboratory Animal (ACCBAL), the Institutional Committees for the Care and Use of Animals (CICUA) of the Pontificia Universidad Javeriana and the Universidad de los Andes and the Research Ethics Committee of the National University of Colombia, 2023.

### ZIKV harvesting and titration

A ZIKV strain from a Colombian patient isolate (GenBank: OP898542.1) was used. This strain was initially passaged in Vero cells (ATCC CCL-81) and then amplified for three rounds in C6/36 HT mosquito cells (ATCC CRL-1660) cultured in L15 medium (Biowest, L0300) supplemented with non-essential amino acids (MEM NEAA, Gibco 1140−050), Tryptose phosphate broth (Gibco 18050−039), and L-Glutamine 2 mM (Biowest, X0550) for 7 days. The virus titers were obtained using the standard plaque assay [30] on BHK-21 cells (ATCC CCL-10). The virus was then stored in aliquots at −80°C until use.

### *In vivo* infection model

Female and male Balb/c mice of 4–5 weeks old were maintained under standard breeding conditions with 12-hour light and dark cycles and provided with water and food *ad libitum*. After 20−22 days of establishing pairs, the 1-day-old offspring were collected for various experiments. For *in vivo* assays, prior to intracerebral (i.c.) inoculation, 5% lidocaine and local cooling (ice for 1 minute) were applied to the injection site which was located at anterior–posterior (AP) 0.5–0.02 mm relative to Bregma. Then, six pups were intracerebrally (i.c.) inoculated with 15–20 µL of ZIKV (4 × 10⁵ PFU/mL), three pups received mock inoculation (supernatant from uninfected C6/36 cells), and three additional pups were left non-inoculated (NI), N = 12 mice. All inoculations were performed in serum-free DMEM. The animals were observed, weighed, and measured daily until 10 days post-infection (dpi). Body length was determined from the nose to the base of the tail using a vernier caliper. Changes in body size (mm day ⁻ ¹) and weight (g day ⁻ ¹) between consecutive days were calculated by dividing the difference between two consecutive measurements by the time interval, providing the average daily growth and weight rate for each time point according to the formula:(Size or weight day X+1−Size or weight day X)/(Day X+1−Day X). Data were obtained from two independent experiments, each first analyzed separately to confirm internal consistency. As both showed similar trends in growth and weight across all treatment groups, the datasets were pooled to increase statistical power and provide a more robust estimate of variability and group effects. Statistical significance was re-evaluated using the combined dataset. The raw data from each independent experiment, as well as the combined dataset, are provided in the data repository (Harvard Dataverse) for full transparency and reproducibility, https://doi.org/10.7910/DVN/GT2BTZ. At the end of the observation period, the pups were euthanized via anesthesia overdose (Ketamine 50 mg/kg, Xylazine 15 mg/kg).

### Tissue processing

At 10 dpi, infected pups (n = 6) and their respective controls (NI and mock-inoculated, n = 3 each) were euthanized, and intracardially perfused with sterile PBS 1X. Brains from three infected animals and one from each control group were immediately extracted and kept on ice. One hemisphere was homogenized under sterile conditions in 500 µL of DMEM, centrifuged at 14,000 rpm and the supernatant was stored at −80°C until use. The other hemisphere was homogenized in 250 µL of sterile PBS, mixed with 250 µL of Trizol® and stored at −80°C. The remaining animals (three ZIKV inoculated and two of each control) were perfused with 4% paraformaldehyde (PFA) after PBS washing; their brains were postfixed in PFA immersion for 48 h and embedded in paraffin. Fixed tissues were processed to obtain 5 µm paraffin sections mounted on poly-L-lysine (100 µg/mL, P8929 Sigma) coated slides. Selected sections were stained with hematoxylin-eosin (H&E) (AB 245880 Abcam). Two independent experiments were performed under identical conditions.

For histological and morphometric analyses, H&E-stained sections from at least two brains per condition (infected and mock) were examined in both independent experiments. All images were obtained using a Zeiss M2 microscope and Zen 2.6 (blue edition) software. Eight representative fields from three sections per brain were captured, each covering an area of approximately 1600 μm^2^. These images were used to quantify the number, area, and circularity of blood vessels using ImageJ software. Vessel circularity was calculated using the formula *Circularity = 4*π × *(area/perimeter²)*, where 1 indicates perfect circularity (maximal dilation) and 0 indicates minimal circularity (normal, non-dilated vessels). Vessels were classified into three categories based on circularity: low (0.1–0.49), intermediate (0.5–0.79), and high (0.8–1). Normal cells, immune-infiltrated cells (IMC), and neurons with nuclear damage (ND) were counted on at least two brain sections per condition (four fields per section) from both independent experiments. Neurons were further classified according to the type of ND observed—pyknosis (chromatin condensation and nuclear shrinkage), karyorrhexis (nuclear fragmentation), or karyolysis (nuclear dissolution); these data, along with the counts of apoptotic cells, were analyzed in the same dataset. Raw data can be found at https://doi.org/10.7910/DVN/GT2BTZ, Harvard Dataverse.

### *In vitro* infection model

For the *in vitro* tests, 3–5 one-day-old pups were euthanized by an overdose of ketamine (15 mg/kg) and xylazine (5 mg/kg). The pups were then washed in alcohol, and under sterile conditions and a stereoscope, their brains were extracted. These brains were processed to obtain cortical neurons, astrocytes, and microglia cells. The procedures for culturing and infecting cells are outlined briefly.

### Cortical neurons culture

For the isolation of cortical neurons, the protocol by Beaudoin et al. [31] was used with some modifications. Brains from 1-day old mice were used (3–5 brains per culture). Under sterile conditions, the brains were extracted, and the cerebral cortices were removed under a stereoscope and placed in dissection medium: Hank’s Balanced Salt Solution (HBSS) supplemented with 0.1% D-glucose, 10 mM HEPES, sodium pyruvate, and antibiotic-antimycotic solution. The tissue was then incubated with 500 µL of 0.25% trypsin solution at 37°C for 20 min. After this time, 1% DNase I was added for 5 minutes at room temperature. The solution was then removed, and the tissue was washed twice with dissection medium and seeding medium: DMEM/F12 supplemented with 10% fetal bovine serum (FBS), antibiotic-antimycotic supplement, 2 mM L-glutamine, and 0.45% D-glucose.

The tissue was disintegrated by mechanical dissociation using fire-polished Pasteur pipettes and the resulting solution was passed through 70-mesh filters and then placed on a 4% bovine serum albumin (BSA) cushion, which was centrifuged at 1000 rpm for 5 minutes at room temperature (2 times). The pellets were collected in seeding medium and centrifuged at 1500 rpm for 5 minutes. Finally, these pellets were resuspended in seeding medium and seeded on culture surfaces (T-75 cm^2^ flasks, 6 or 24 well plaques, or glass slides) pre-coated with a cell adhesion matrix (Collagen I and poly-L-lysine in PBS), and incubated for 1 hour at 37°C. After this incubation period, the medium was replaced with maintenance medium: Neurobasal medium supplemented with B-27^TM^, L-glutamine, and antibiotic-antimycotic. After 24 hours post-seeding (hps), the medium was removed, the cultures were PBS washed, and fresh maintenance medium supplemented with 2.5 µM Cytosine β-D-arabinofuranoside (AraC) was added for 48 hours to purify the culture. Following this, the medium was removed, the cultures were washed with PBS, and fresh maintenance medium was added.

### Astrocytes and microglia cultures

The protocol by Velandia et al [32]. was used with some modifications for the isolation and culture of mixed glial cells. Brains from 1-day-old mice were used (1 brain per T-75 cm^2^ culture flask). The brains (without cerebellum) were placed in digestion solution (collagenase, dispase, DNAse I, L-cysteine, and L-glutamine in DMEM/F12) for 20 minutes at 37°C. Mechanical dissociation of the tissue was performed by pipetting. The supernatant was discarded and replaced with 1.5 mL of ovomucoid solution (L-Glutamine, DNase I, BSA, and trypsin inhibitor in DMEM/F12), in which the tissue was homogenized and incubated again for 2 minutes at 37°C. The cell suspension was centrifuged for 3 minutes at 1600 rpm; the supernatant was discarded, and 1.5 mL of ovomucoid solution was added again. Next, 4 mL of culture medium (DMEM/F12 supplemented with 10% FBS, 0.2 mM L-glutamine, and antibiotic-antimycotic) was added, and the suspension was centrifuged for 5 minutes at 1600 rpm. The supernatant was discarded; the cells were resuspended in culture medium, seeded into T-75 cm^2^ culture flasks, and incubated at 37°C with 7% CO₂. Growth was monitored for each cell type. Astrocyte cultures were typically purified by day 10 after seeding, while microglial cells were recovered between 18 and 25 days after seeding and to enhance microglial cell production.

The purification process for both cultures was performed following the protocol from Skaper et al., with a few modifications [33]. It began with cultures constant shaking at 200 rpm for 2 hat 37°C. The medium was then removed and centrifuged at 1500 rpm for 5 minutes. The resulting pellets, primarily consisting of superficial microglial cells, were resuspended in fresh culture medium and mixed glial cell-conditioned medium (1:1) and seeded in 24 or 96-well plaques or glass slides covered with Poly-L lysine (10 µg). The remaining cells in the initial culture were again placed in constant shaking at 200 rpm for 18 h at 37°C to detach remaining microglial cells and debris, whose were discarded. These cultures (composed mainly of astrocytes) were washed, and fresh culture medium was added. The cultures were maintained for an additional 8 days to allow enrichment with mature astrocytes.

### Cell culture infection

Astrocytes, microglial cells, and neurons were seeded at different densities depending on the plate format: 10,000 astrocytes, 30,000 microglial cells, or 50,000 neurons per well in 96-well plates; 20,000 astrocytes, 60,000 microglial cells, or 100,000 neurons per well in 24-well plates; and 100,000 astrocytes, 300,000 microglial cells, or 500,000 neurons per well in 6-well plates. At 24 h post-seeding, cells were infected with ZIKV at multiplicities of infection (MOI) ranging from 0.01 to 3.The viral inoculum was diluted in the respective culture medium supplemented with 2% FBS and incubated with the cells for 1 hour at 37 °C in half of the standard medium volume used to maintain the cultures (60 µL for 96-well plates, 250 µL for 24-well plates, and 1 mL for 6-well plates). After this period, the inoculum was removed and replaced with fresh culture medium (final volumes of 100 µL, 500 µL, and 2 mL, respectively), and the cells were maintained for 24-, 48-, or 72-hours post-infection (hpi).

### Cell death evaluation

Cultured astrocytes (10,000), microglia (30,000) and neurons (50,000) seeded in 96-well plates were infected with ZIKV at MOI ranging from 0.01 to 3 and maintained for 24, 48, or 72 hpi. Then, cells were washed and incubated at 37°C during 45 min with Calcein-AM (2,5 µM, Invitrogen) and Ethidium Bromide (4 µM). After this time, cells were washed and kept at 37°C for additional 15 min. Finally, cell fluorescence was measured using the ClarioStar (BMG Labtech) and analyzed using the MARS 3.41 software. All viability assays were performed in three independent biological experiments, each containing triplicate technical replicates per condition. Data inclusion required technical consistency across replicates and biological plausibility among independent experiments. One biological replicate was excluded from the pooled analysis and due to ethical reasons related to animal use, the excluded experiment was not repeated.

### RNA extraction, amplification of cellular transcripts

Astrocytes (100,000 cells), microglia (300,000 cells) and neurons (500,000 cells), were seeded in 6-well plates and infected (as described above) and maintained in 1 mL of culture medium during infection. After infection, cultures were incubated in 2 mL of medium for 24-, 48- and 72 hpi. At these post-infection time points, infected and non-infected control cultures (including all remaining cells and the entire culture volume from each well), as well as brains from infected (three half-brains) and uninfected animals (one half-brain per control), were collected from two independent experiments. All samples were processed with TRIzol® reagent according to the manufacturer’s instructions.

For primary cultures and brain samples, RNA was extracted from two independent experiments. Each experiment consisted of pooled material (three wells per condition for cell cultures and three half-brains per group for brain samples). RT-qPCR reactions were performed in duplicate using 100 ng of total RNA from each independent experiment, employing the Luna® Universal One-Step RT-qPCR Kit (NEB #E3005) with SYBR Green and specific primers for cellular transcripts (TNF-α, IL-6, IL-10, IFN-β, MCP-1, PKR, and STAT-1). Amplification conditions were as follows: 95 °C for 10 min, followed by 40 cycles of 95 °C for 30 s and 60 °C for 1 min. β-actin was used as the internal reference for relative quantification. Melting curve analysis (95 °C for 15 s, 60 °C for 15 s, and 95 °C for 15 s) confirmed amplicon specificity. Data were analyzed using the Schefe method [34], and fold changes are presented as log-transformed values for improved visualization.

### Quantification of viral RNA

For ZIKV RNA quantification in primary cultures, three independent experiments were performed, each consisting of pooled material from three wells per condition, analyzed in duplicate. This third experiment was included to increase the statistical power and to confirm the reproducibility of the results obtained in the initial two experiments. The primers and specific ZIKV probe have been previously described [35]. A ZIKV standard curve was established using TaqMan primers and probe (IDT). Briefly, 10-fold serial dilutions of ZIKV genetic material from BEI Resources (NR-50244) were used to correlate the cycle threshold (Ct) value with the number of molecules/µL of viral RNA. The RNA copy number (molecules/µL) was calculated using the formula: [RNA concentration (µg/mL)]/ [RNA transcript length (nucleotides) × molecular weight of a nucleotide (330 Da) × Avogadro’s number (6.023 × 10^23^)]. The RT-qPCR for viral RNA was conducted under the following cycling conditions: 95°C for 10 minutes for pre-denaturation, followed by 40 cycles of 95°C for 15 seconds and 60°C for 1 minute.

### Immunofluorescence of whole brains and cell cultures

Before the immunofluorescence (IF), the brain sections were processed as follows: after dewaxing by heat (56°C for 2 h) and 100% xylol (2 times for 15 minutes), the tissues were hydrated with a descending gradient of ethanol (100%, 90%, 80%, 70%) for 15 minutes each and finally with PBS for 15 minutes. Finally, the tissue sections were incubated for 5 minutes in 10 mM sodium citrate (pH 6.0) previously heated at 90°C, and washed with 1X PBS, before the IF procedure. On the other hand, 20,000 astrocytes, 60,000 microglial cells, or 100,000 neurons were seeded on glass slides and fixed with 4% PFA for 30 minutes, washed, and stored in sterile PBS at 4°C.

For IF, both cell cultures and brain sections were incubated with ammonium chloride (50 mM) for 30 minutes, washed with PBS, and permeabilized with 0.3% Triton X-100. Blocking of non-specific sites was performed using 10% goat serum. Primary antibodies against the C protein of the virus (NBP3–13200, Novus) and/or cellular markers for astrocytes: Glutamine Synthase (GS, sc-74430, Santa Cruz), and Glial acidic fibrillary protein (GFAP, M0761, DAKO); for neurons: Collapsin Response Mediator Protein 2 (CRMP-2, #35672, Cell Signaling) and Microtubule Associated Protein 2 (MAP2, #8707, Cell signaling); and for microglia CD11b (MA5–17858, eBioscience), Transmembrane protein 119 (TMEM119, #90840, Cell signaling) and Orexin 2 Receptor (OX2R, sc-14392, Santa Cruz) were diluted in PBS containing 0.3% BSA and 0.3% Triton X-100 and incubated overnight at 4°C.

Subsequently, the respective secondary antibodies were applied; Alexa 488 and Alexa 594 conjugated secondary antibodies were used for cultures (A-11008 and A-11012, Thermo Fisher), while biotin-conjugated secondary antibodies were used for tissues (BA-1000–1.5, or BA-9200–1.5, VectorLabs). These were then incubated with streptavidin conjugated to FITC (SA-5001–1, VectorLabs) or Alexa-Fluor 594 (S11227, Thermo Fisher). Finally, nuclei were counterstained with DAPI (1:2500 for tissues, 1:5000 for cells, B2261, Sigma-Merck), and sections were mounted with ProLong Gold antifade (#9071, Cell signaling). Observations were made under a Zeiss M2 microscope, coupled to the Colibri 7 fluorescence system and Apotome 2 deconvolution system. Images were captured using the Zen 2.6 (blue edition) software.

Infection percentages from brain sections and primary cultures were calculated by counting the total of positive cells for both the ZIKV capsid protein and their respective population markers (GFAP for astrocytes, CD11b for microglia, and MAP2 for neurons). Analyses were performed in four fields from three sections per brain, using two brains per experiment in two independent experiments. Cell quantification was carried out using the ImageJ plugin. Raw data counts can be found in https://doi.org/10.7910/DVN/GT2BTZ, Harvard Dataverse.

### Evaluation of viral production

Twenty thousand A549 cells were seeded in 96-well plates and maintained in DMEM supplemented with 2% FBS and antibiotic-antimycotic. Brain homogenates or supernatants from neurons, astrocytes, or microglia cells were centrifuged at 10,000 *xg* for 5 minutes, and the resulting clarified supernatants (50 µL) were added to A549 cell monolayers and incubated for 72 h at 37°C and 5% CO₂. Pooled brain homogenate samples were obtained from three half-brains per experiment (two independent experiments). The MOI was calculated prior to animal inoculation to ensure consistent infection conditions; in this assay, however, the objective was to determine whether brain-derived material contained infectious virus capable of transmitting infection, rather than to establish an exact MOI after cell infection.

After incubation, the cells were fixed with 4% PFA and processed processed for ZIKV detection by immunoperoxidase staining. Cells were permeabilized with 0.3% Triton X-100 for 30 minutes, washed with PBS, and incubated with 50% methanol and 0.25% H₂O₂ in PBS for 30 minutes to block endogenous peroxidases. Then, cells were PBS washed, blocked with 10% goat serum for 1 hour, and incubated with the specific antibody for ZIKV capsid protein overnight at 4°C. Next, cells were washed with PBS and incubated at room temperature with the secondary antibody (KPL α-rabbit-peroxidase-labeled, 5220–0336 (074–1506), SeraCare) for 1 hour. Finally, the revealing solution consisting of diaminobenzidine (DAB, 0.075%) and H₂O₂ (0.02%) diluted in Tris-HCl, 0.1M pH 7.2, was added to the cells, and the cell staining was analyzed under the inverted microscope Zeiss Axiovert A1, and images were captured using Zen 3.1 software (Zeiss).

Infected cells were counted using the Fiji/ImageJ software. Manual quantification was performed using the cell counter tool, and the data was confirmed using the infection counter plugin for automated quantification of the infected cells [36]. The total number of infected cells present in three wells (8 fields per well) of two independent experiments was used to calculate the percentage of cell infection. Raw data counts can be found in https://doi.org/10.7910/DVN/GT2BTZ, Harvard Dataverse.

### Statistical analysis

Statistical analyses were performed using GraphPad Prism software (10.6.1). As controls, cells and animals were inoculated with mock (supernatants from non-infected C6/36-HT mosquito cells). All data were tested for normality using the Shapiro–Wilk test (*p > 0.05*). For growth and weight analysis two independent experiments were carried-out, each with 12 pups (3 NI, 3 Mock-inoculated and 6 ZIKV-infected). After calculating daily growth and weight rates as described above, data normality was assessed, and either a one-way ANOVA or Kruskal–Wallis test was performed depending on data distribution. Post-hoc pairwise comparisons were conducted using Dunnet’s or Dunn’s multiple comparison test, respectively. Results are expressed as mean ± standard deviation (SD), and differences were considered significant when *p < 0.05*.

For vascular morphometry (vessel area), quantification of normal cells, ND, and IMC, as well as comparisons of ND subtypes between mock-inoculated and ZIKV-infected groups, were performed in two independent experiments conducted under identical conditions, both showing the same trend. Therefore, data were pooled to increase statistical power. Comparisons between groups were performed using the non-parametric Mann–Whitney U test. When one group contained only zero values, normality could not be assessed and the Mann–Whitney U test was applied. Differences were considered statistically significant at *p* < 0.05.

Viability assays were performed in three independent biological experiments, each containing triplicate technical replicates per condition. One of these experiments was excluded from the pooled analysis because its results showed erratic values (including unusually high cell mortality at low MOIs, inconsistent trends across post-infection times, and poor reproducibility among technical replicates) which did not follow the consistent trend observed in the other two experiments performed under identical conditions. The remaining two experiments were therefore combined and analyzed using one-way ANOVA (p < 0.05) followed by Dunnett’s multiple comparison test. All raw data, including those from the excluded experiment, are publicly available in the Harvard Dataverse repository https://doi.org/10.7910/DVN/GT2BTZ.

For viral quantification by RT-qPCR, two independent experiments were performed in duplicate for brain lysates, whereas three independent experiments (also analyzed in duplicate) were conducted for *in vitro* cultures, using both lysates and supernatants. Comparisons were made using either Kruskal–Wallis or one-way ANOVA tests, followed by Dunn’s or Dunnett’s post-hoc analyses, respectively. Differences were considered statistically significant when p < 0.05.

## Results

### *In vivo* model description

**Fig 1:**
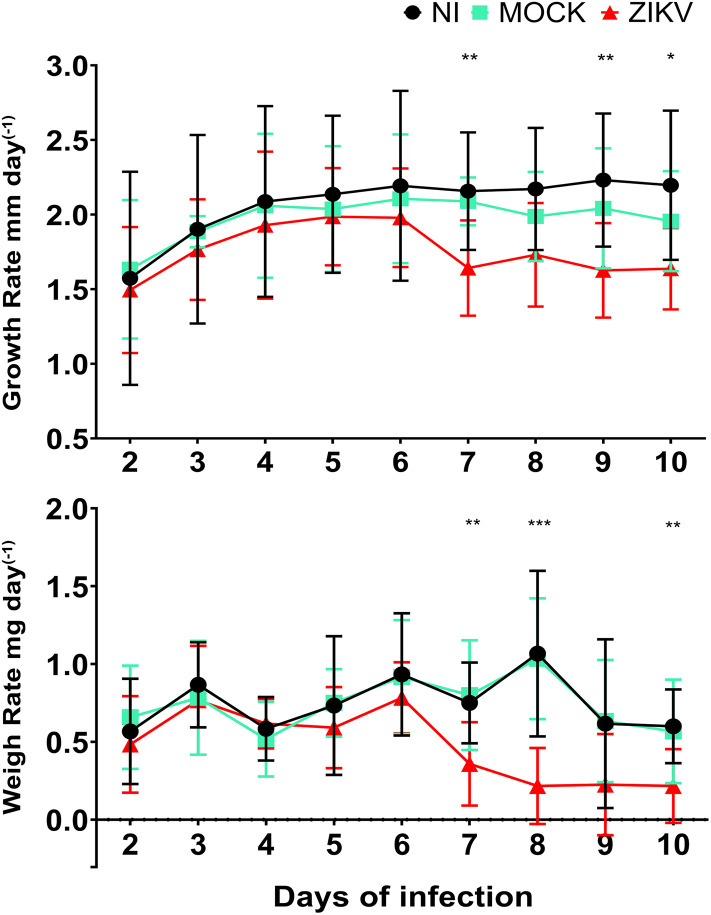
Growth and weight assessment of i.c. infected mice and controls. Body length (nose-to-tail base) and weight of ZIKV-infected, mock-inoculated, and NI mice were recorded daily for 10 dpi using a vernier caliper and a precision balance, respectively. Data were obtained from 6 ZIKV-infected, 3 mock-inoculated, and 3 NI mice (N = 12) across two independent experiments. Values represent the rate of change between consecutive days, with day 1 set to zero as no prior measurement was available. Depending on data distribution, one-way ANOVA or Kruskal–Wallis tests were applied, followed by Dunnett’s or Dunn’s post-hoc comparisons, respectively. Results are expressed as mean ± SD. Statistical significance was set at *p *< 0.05*, **p < 0.01 and ***p < 0.001.

In the mice model, the ZIKV strain (isolated from a Colombian patient) did not induce a lethal infection, allowing the neonates to survive until day 10 post-infection. Following i.c inoculation, no mortality was observed in any of the experimental groups, and none of the infected animals showed any of the severe signs described in the Animal Welfare section; therefore, the animals were kept alive until day 10 post-infection. However, the virus induced the following neurological symptoms: mild inactivity, ataxia, and axial weakness, which became evident by the 7^th^ dpi and progressed to paralysis, loss of balance, and prostration by day 10. A reduction in growth and weight rates (Fig 1) was observed between days 7 and 10 dpi compared to the control group.

Then, at 10 dpi animals were euthanized and brains were collected. A first half of the brains were processed for RNA extraction and the other half were used to evaluate the virus’s infectious capacity. A low RNA viral load of 6 × 10^3^ copies was detected; however, that brain lysate caused infection in approximately 70% of A549 cells (S1 Fig, Raw counts can be found at Harvard Dataverse: https://doi.org/10.7910/DVN/GT2BTZ.. This indicates that despite the low amount of viral RNA obtained from the brain, the virus can readily infect highly susceptible cells as the A549 cell line.

**Fig 2:**
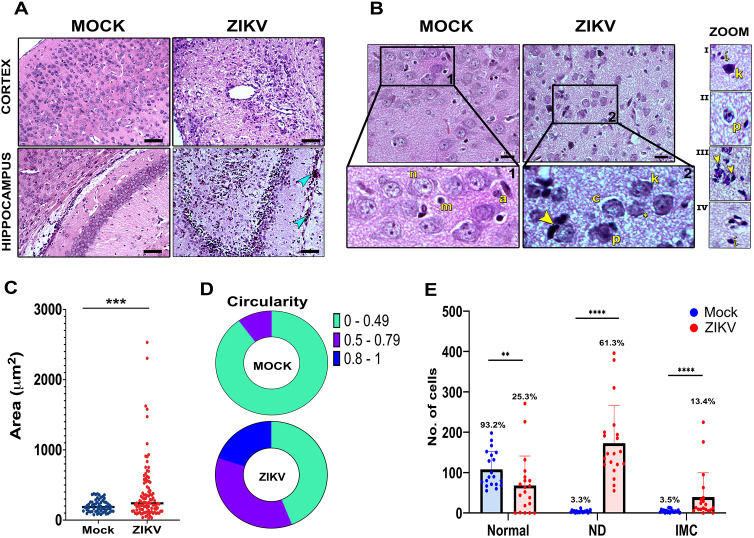
Histological and morphometric evaluation of brains from i.c ZIKV-infected and control mice (A) Histological sections (5 µm) of brains from 10 dpi Mock-inoculated or ZIKV-infected mice stained with H&E. Panoramic images of mock brains show well-defined cortical stratification and clearly distinguishable molecular and granular layers in the hippocampus. In contrast, ZIKV-infected brains exhibited cortical alterations with dilated blood vessels, and the hippocampus showed nuclear layer disorganization and immune cell infiltration (outlined blue arrowhead). Scale bars = 50 µm. (B) Higher magnification views show healthy cortical neurons (n), microglia (m), and astrocytes (a) in control animals. In infected brains, extensive parenchymal damage was observed, including neuronal damage (ND), inflammatory infiltrates **(i)**, and neurons with nuclear alterations (i–vi) like karyorrhexis **(k)**, pyknosis **(p)**, karyolysis **(c)**, faint or pale nuclei (*), satellite glial cells (arrowhead), capillary collapse and neuropil disruption. Scale bars = 20 µm. **(C–D)** Capillary and blood vessel area and circularity were quantified from eight fields per brain (three sections per animal, in two independent experiments) using Fiji/ImageJ. **(E)** Normal cells, ND, and IMC were quantified from four fields (two sections per brain, in two independent experiments). The number of cells per condition is shown, as well as the equivalent percentage. Statistical comparisons were performed using the Mann–Whitney U test (**p < 0.01, ***p < 0.001, ****p < 0.0001).

Histological evaluation of brain tissue from the control group (Mock) revealed normal cortical morphology, with clear stratification and small capillaries. Similarly, in the CA1 region of the hippocampus, the molecular and granular layers were well defined. In contrast, brain tissue from ZIKV-infected animals showed markedly dilated blood vessels and abundant inflammatory infiltration in the cortex, while in the hippocampus, structural damage and immune infiltration were evident -outlined blue arrowhead- (Fig 2A). A closer inspection of the brain parenchyma in mock-inoculated animals revealed large neurons (n) with well-defined cytoplasm, central nuclei with visible nucleoli, as well as inactive glial cells: microglia (m) and astrocytes (a), -Fig 2B-. In contrast, the cortex of infected animals displayed basophilic neurons with pyknotic (p) or absent nuclei (karyolysis, c), suggesting necrosis (Fig 2B, inset). Glial cells were frequently observed surrounding degenerated neurons (yellow arrowhead), possibly indicating neuronophagy. These neurons showed condensed chromatin and scant cytoplasm (*). Nuclear fragmentation was also observed, consistent with pyknosis (p) and karyorrhexis (k). Additional alterations in the tissue included capillary collapse, neuropil vacuolization, and immune cell infiltration -i- (insets, Fig 2B i–iv).

Given the prominence of these findings, the area and circularity of vessels and capillaries were quantified. In Mock animals, the mean vessel area was approximately 202 µm^2^ (Fig 2C), and 90% of vessels displayed circularity values between 0.1 and 0.49, consistent with non-dilated, regular, and tortuous morphologies (Fig 2D). In contrast, in infected animals, the mean vessel area increased significantly to 381 µm^2^, suggesting abnormal angiogenesis and vascular dilation (Fig 2C). Regarding circularity, 20% of vessels showed values close to 1, indicating highly dilated and rounded morphologies, while 36% exhibited circularity values between 0.5 and 0.79, consistent with moderate dilation and irregularity (Fig 2D).

Similarly, ND was quantified in the cortices of infected animals, revealing a significant increase in nuclei showing pyknosis, karyorrhexis, and karyolysis compared with healthy cells (Figs 2E, S2). A significant increase in IMC was also observed, with lymphocytes and macrophages being the most frequent (Fig 2E).

**Fig 3:**
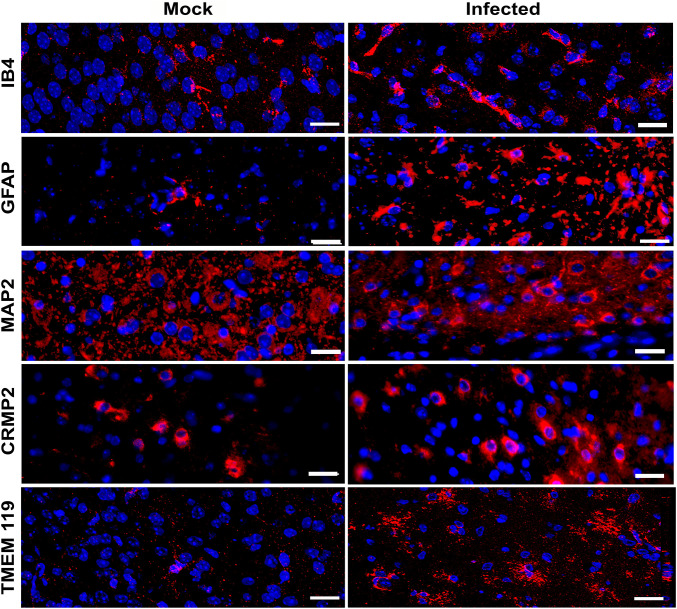
IF evaluation of different cell populations in brains of infected mice. Brain sections from ZIKV-infected mice were collected at 10 dpi and subjected to IF staining. Capillaries were labeled with IB4, astrocytes with GFAP, microglia with TMEM119, and neurons with MAP2 and CRMP2. Nuclei were counterstained with DAPI. Representative images of the cerebral cortex from mock-inoculated and ZIKV-infected mice, derived from two independent experiments, are shown. Scale bars: IB4, GFAP, MAP2: 20 µm; CRMP2: 50 µm; TMEM119: 10 µm.

A more comprehensive evaluation of cell populations in the tissue sections of infected animals (using the IF technique), confirmed an increase in capillaries in all brain regions, as evidenced by labeling with the lectin IB4. Activation of cells, highly positive for GFAP and TMEM119 (astrocytes and glial cells respectively) were associated with cellular hypertrophy and elongation of the cytoplasmic processes (Fig 3). After evaluating the integrity of cerebellum or cortex neuronal soma and axons or dendrites, using MAP2 and CRMP2 markers, no obvious changes were observed (Fig 3).

**Fig 4:**
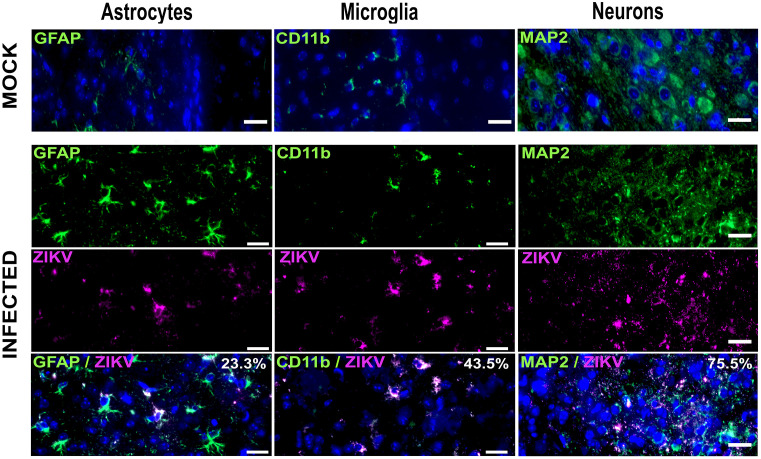
ZIKV capsid protein detection in brain sections of i.c. infected mice. Brain tissue sections from mice at 10 dpi were processed for IF staining. Sections were incubated with antibodies against the ZIKV capsid protein along with cell type–specific markers for microglia (CD11b), astrocytes (GFAP), and neurons (MAP2). Nuclei were counterstained with DAPI. Analyses were performed on four fields from three sections per brain, using two brains per experiment in two independent experiments. Representative images of the cerebral cortex from mock-inoculated and ZIKV-infected mice are shown. Scale bars: GFAP, CD11b, MAP2, 20 µm.

Additionally, numerous microglial cells (43.5%) and neurons (75.5%) in the cortex, were positive for ZIKV, whereas only a few activated astrocytes (23.3%) showed viral antigen positivity (Fig 4). Raw data counts can be found at Harvard Dataverse: https://doi.org/10.7910/DVN/GT2BTZ

Given that in an *in vivo* model is challenging to precisely identify which cell populations are responding and to what extent, we used *in vitro* approaches to evaluate the response patterns of three key central nervous system cell types that play crucial roles in ZIKV pathogenesis and the establishment of the local immune response.

### Maturation status and characterization of cultured neurons, astrocytes, and microglia from neonatal mice

Cultures of cortical neurons, astrocytes, and microglia were established from 1-day-old mice and maintained for 7–20 days post-seeding, depending on the cell type, achieving 95–98% purity in each culture, ensuring that the observed response corresponded to a single cell type. Culture purity raw data counts can be found at https://doi.org/10.7910/DVN/GT2BTZ, Harvard Dataverse.

**Fig 5:**
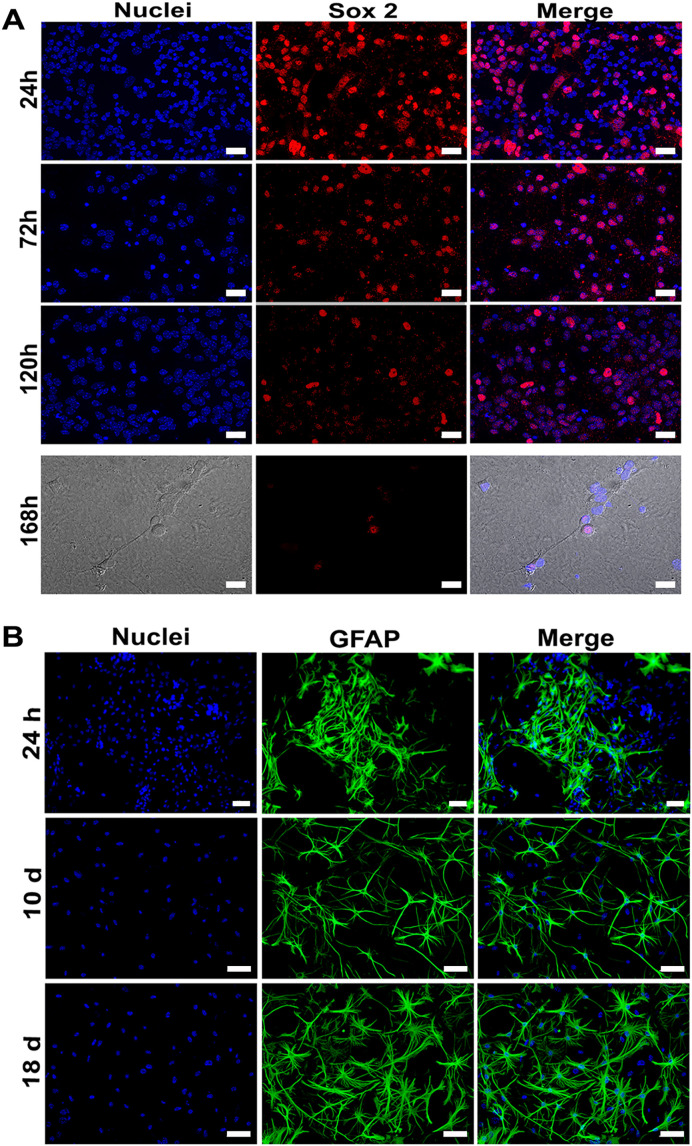
Time-course analysis of neuronal and astrocyte marker expression in primary cultures. A) Primary neuronal cultures were analyzed at 24, 72, 120, and 168 hps for SOX2 protein expression by IF. B) Primary astrocyte cultures were analyzed at 24 h, 10 days, and 18 days post-seeding for GFAP protein expression. Cells were fixed at each time point, incubated with the respective primary antibodies, and visualized with appropriate fluorescent secondary antibodies. Nuclei were counterstained with DAPI. Representative images from three independent experiments are shown. Scale bars: A, 20 µm; B, 50 µm.

**Fig 6:**
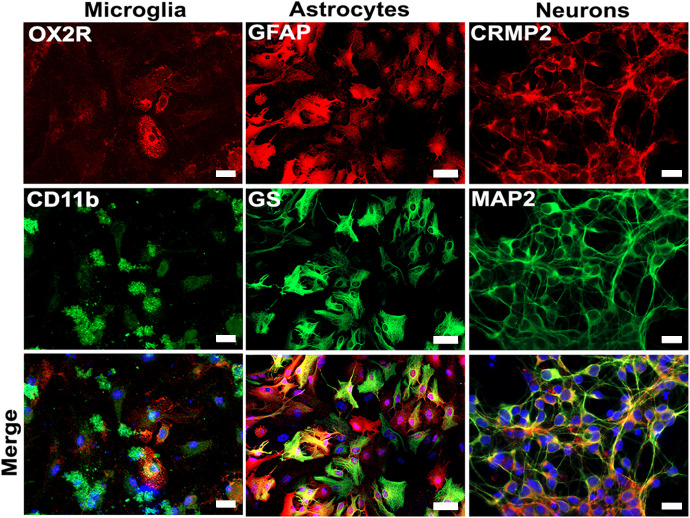
Co-expression of different cell-type–specific markers in isolated primary neural cell cultures. Microglial cells were isolated from mixed glial cultures and stained for CD11b and OX2R. Astrocytes were stained for GFAP and GS. Neurons were stained for MAP2 and CRMP-2. Cell-type–specific markers were detected using corresponding primary antibodies and appropriate secondary antibodies conjugated to fluorescent dyes. Nuclei were counterstained with DAPI. Representative images from three independent cultures are shown. Scale bars: 20 µm (microglia and neurons); 50 µm (astrocytes).

In neuronal cultures, a significant reduction in the expression of the immature cell marker Sox2 was observed over time (Fig 5A). At 120 hours post-seeding (hps), less than 5% of the cells were positive for Sox2. By 168 hps, Sox2 was primarily detected in the nuclei of non-neuronal cells (Fig 5A). At this time point, cortical neurons exhibited typical morphology with small somas and numerous cytoplasmic processes, which were positive for CRMP-2 and MAP-2 (Fig 6).

Astrocyte cultures demonstrated a maturation dynamic characterized by morphological changes and increased expression of the GFAP protein. Initially, only a few cells were positive for GFAP and exhibited a slender morphology, forming niches (Fig 5B). By 10 days post-seeding, most cells displayed a stellate morphology and showed strong GFAP immunoreactivity. At 18 days post-seeding, nearly all astrocytes exhibited robust GFAP expression, exhibiting a hypertrophic, stellate morphology with long cytoplasmic processes (Fig 5B). Most of these cells were also expressing glutamine synthetase (GS) (Fig 6), indirectly indicating the cell’s active metabolism.

In the case of microglia, when growing on astrocyte cultures, they showed a rounded morphology (S3 Fig). Upon collection and reseeding on slides, these cells expressed OX2R and CD11b (Fig 6). With the differentiation status of the cultures characterized and defined, we proceeded to evaluate their susceptibility to ZIKV infection and their response to the virus.

### Postnatal cortical neurons were highly susceptible to ZIKV infection

To determine whether terminally differentiated cortical neurons, astrocytes, and microglia cells were infected by and respond to ZIKV infection, we evaluated the susceptibility and impact of the virus on the survival of each cell population at different MOI (0.01, 0.1, 0.5, 1, 3) and post-infection times (24, 48, and 72 h).

**Fig 7:**
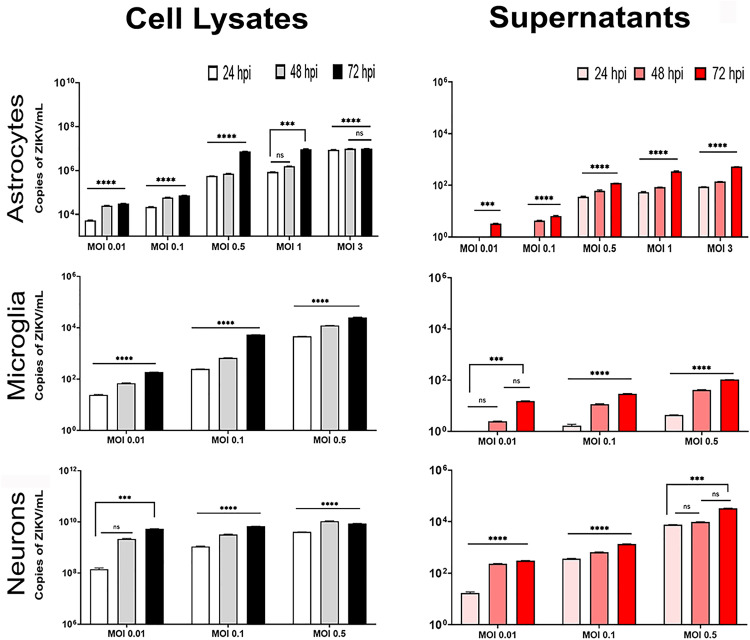
Quantification of intracellular and released viral RNA in ZIKV-infected primary neural cell cultures. Primary astrocytes, microglia, and neurons were infected with ZIKV at different MOIs (0.01–3) and incubated for 24, 48, or 72 hpi. At each time point, culture supernatants and whole-cell lysates were collected separately. Viral RNA was extracted using TRIzol® and quantified by RT-qPCR. Data are expressed as viral copies per milliliter (copies/mL) and presented as log₁₀-transformed values for improved visualization. Results represent the mean ± SD from three independent experiments, each performed in duplicate. Statistical significance was determined using one-way ANOVA or Kruskal–Wallis tests, followed by Dunnett’s or Dunn’s multiple comparison tests, respectively, ***p < 0.001, ****p < 0.0001, ns: not significant.

We found that all three cell types were permissive to ZIKV and produced infectious particles (S4 Fig). Astrocytes were infected at the lowest MOI (0.01 and 0.1), showing between 1.73x10^3^ to 2.1x10^4^ viral copies/mL at 24- and 48-hours post-infection (hpi), respectively, and increasing to approximately 6x10^6^ viral copies/mL at a MOI of 0.5. Using a MOI of 1 and 3, the number of copies reached 1x10^7^ regardless of the time point. Viral RNA copy numbers in the supernatants were low, with values of less than 10 at a MOI of 0.01 at 72 hpi, gradually increasing to 100 and 1000 copies/mL at MOIs of 0.5, 1, and 3, respectively (Fig 7).

In microglial cells, the number of viral RNA copies/mL ranged between 10–100 at a MOI of 0.01 at 24- and 48- hpi), respectively. This number increased with time and MOI, reaching a maximum value of 1x10^4^ copies/mL at 72 hpi. Interestingly, the release of the virus into the medium from these cells was very low, with fewer than 50 copies/mL at all-time points and MOI of 0.01 and 0.1, peaking at 150 copies/mL at 72 hpi (Fig 7). In contrast, cortical neurons exhibited high susceptibility to the virus, producing the highest number of intracellular viral RNA copies, which varied according to the MOI and time. The lowest number of copies/mL (1x10^8^) was obtained at 24 hpi with a MOI of 0.01, increasing to 1x10^9^ copies/mL at both 48 and 72 hpi. With MOI of 0.1 and 0.5, a slight increase in the number of copies/mL (1x10^9^ to 1x10^10^) was observed at 48 and 72 hpi respectively. Similarly, the virus produced and released into the supernatant showed the lowest number of copies/mL (10 copies) at 24 hpi with an MOI of 0.01, and the highest values (1x10^4^) at 72 hpi with an MOI of 0.5 (Fig 7).

**Fig 8:**
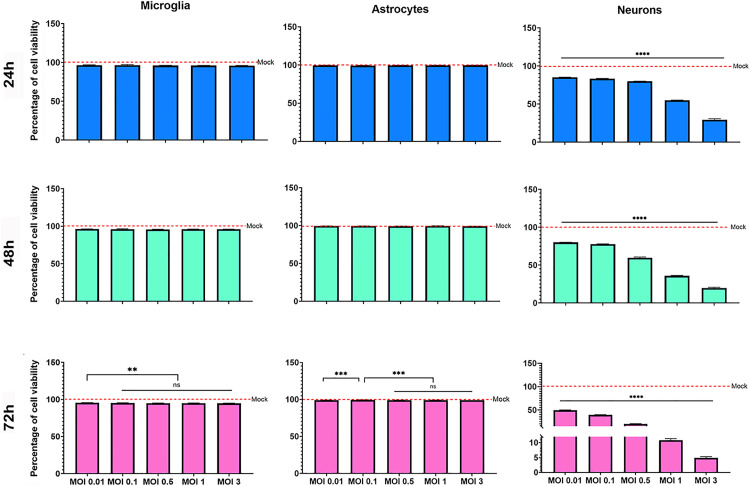
Evaluation of cell viability in ZIKV-infected primary glial and neuronal cultures. Primary microglia, astrocytes, and neurons were infected with ZIKV at different MOI (0.01–3) and incubated for 24, 48, or 72 hpi. At each time point, cell viability was assessed using Calcein-AM staining. Data are presented as the percentage of viable cells relative to mock-inoculated controls. Results represent the mean ± SD from two independent experiments, each performed in triplicate. Statistical significance was determined using one-way ANOVA followed by Dunnett’s multiple comparisons test as post hoc test, **p < 0.01, ***p < 0.001, ****p < 0.0001, ns: not significant.

Regarding the survival of these cell populations, we found that astrocytes and microglia viability is maintained over the post-infection times (24, 48 or 72 hpi) and exhibited very low levels of cell death following infection (approximately 0.3% and 4%, respectively) with no significant variation across either MOI (Fig 8). In contrast, neuronal survival was clearly dependent on both MOI and time post-infection. At the lowest MOIs (0.01 and 0.1), neuronal cell viability decreased progressively over time, with cell death ranging from 15–24% at 24–48 hpi to 50–60% at 72 hpi. At higher MOIs (1 and 3), neuronal death was already 55–75% at 24 hpi and reached 100% by 72 hpi (Fig 8). Importantly, viability assays were performed in three independent experiments, each containing triplicate technical replicates per condition. One of the three experiments was excluded from the pooled analysis due to inconsistencies with the other two independent replicates. Nevertheless, all raw data, including those from the excluded experiment, are available at Harvard Dataverse: https://doi.org/10.7910/DVN/GT2BTZ.

### ZIKV infection induced an increase in the expression of inflammatory mediators

**Fig 9:**
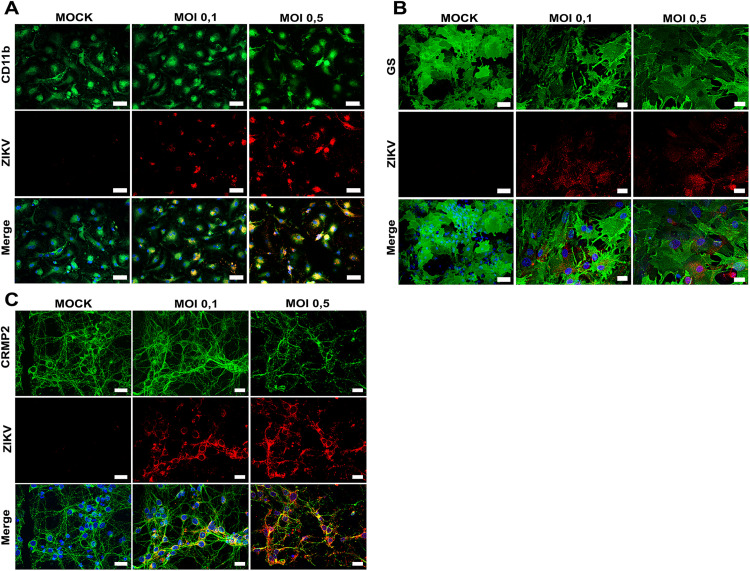
Detection of ZIKV infection in primary astrocytes, microglia, and neurons by IF. (A) Primary microglia, (B) astrocytes, and (C) neurons were infected with ZIKV at MOIs of 0.1 or 0.5 and maintained in culture for 48 hpi. Then, cells were fixed and incubated with an antibody against ZIKV capsid protein (red) together with cell-type–specific markers (green) to confirm the identity of each population. Nuclei were counterstained with DAPI (blue). Representative images from three independent experiments performed in duplicate. Scale bar: 20 µm.

Based on the results of survival and viral production *in vitro*, we evaluated the expression of immune response-related molecules in three cell populations at 48 hpi with MOI of 0.1 and 0.5. We initially confirmed cell infection by detecting viral antigens through IF, finding that all cell types were susceptible to the virus (Fig 9).

Then, we evaluated the transcription of markers associated with the immune response, including TNF-α, IL-6, the chemokine MCP-1, and the anti-inflammatory interleukin IL-10, as well as molecules related to the IFN-β-mediated pathway, such as PKR and STAT-1, both *in vitro* (cell cultures) and compare it to the results of the *in vivo* model (brain lysates).

**Fig 10:**
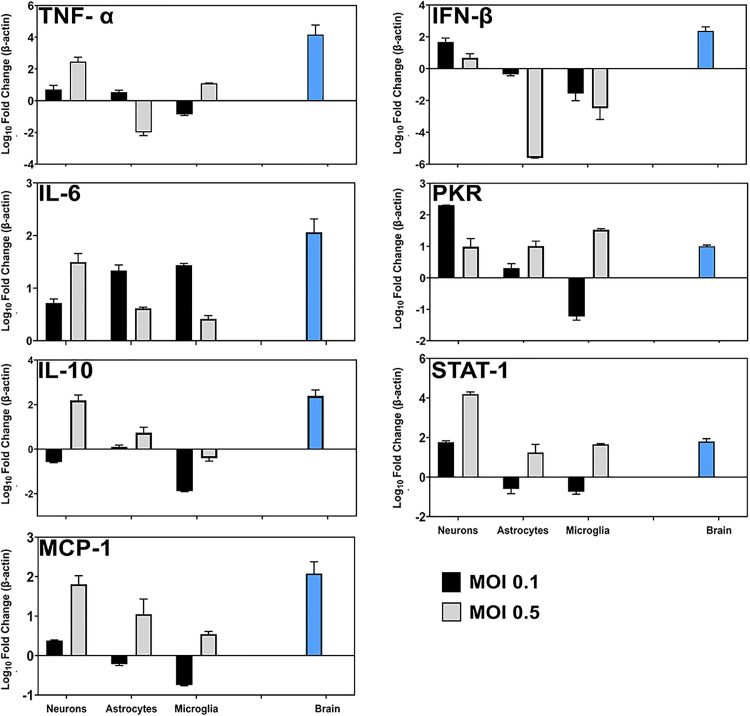
Evaluation of immune mediator expression in brains and primary neural cell cultures following ZIKV infection. Half-brains from ZIKV-infected (n = 3) and mock-inoculated animals (n = 1) were collected at 10 dpi from two independent experiments. In parallel, primary microglia, astrocytes, and neurons were infected with ZIKV at MOIs of 0.1 or 0.5 and harvested at 48 hpi (two independent experiments; each sample consisted of pooled material from three wells per condition). Total RNA was extracted, and TNF-α, IL-6, IL-10, MCP-1, IFN-β, PKR, and STAT-1 expression levels were quantified by RT-qPCR. Fold-change values were calculated using the Schefe method [34], normalized to β-actin, and log₁₀-transformed for visualization. Data represent mean ± SD from two independent biological experiments (each performed in duplicate). Given the limited number of replicates, results are presented descriptively without statistical analysis.

*In vitro*, when analyzing the expression of immune mediators, astrocytes and microglia showed distinct and MOI-dependent profiles. At an MOI of 0.1, both astrocytes and microglia (as central coordinators of the neural tissue response to injury) strongly upregulated IL-6 expression, although only astrocytes showed a slight increase in TNF-α. Both cell types displayed mild deregulation of IL-10 and MCP-1, as well as of IFN-β and its downstream molecules PKR and STAT-1. When the MOI was increased to 0.5, microglia exhibited a slight increase in TNF-α and MCP-1 expression, while astrocytes showed a more pronounced induction of MCP-1. Interestingly, both cell types showed a reduction in IL-6, IL-10, and IFN-β expression at this higher MOI, with this decrease being more marked in astrocytes. In contrast, PKR and STAT-1 were upregulated in both cell types under these conditions (Fig 10).

Interestingly, infected cortical neurons overexpressed various markers at both MOI. At an MOI of 0.1, neurons significantly overexpressed IFN-β, STAT-1, and PKR while downregulating IL-10. When infected with the highest MOI, neurons exhibited higher expression levels of TNF-α, IL-6, IL-10, MCP-1, and STAT-1, while PKR and IFN-β were downregulated (Fig 10). These results could demonstrate that neurons are the primary producers of inflammatory mediators during ZIKV infection.

*In vivo*, at 10 dpi, despite the low amount of quantified viral RNA, a robust pro-inflammatory response was found in the brains of infected animals, as indicated by increased expression of TNF-α (4.5 times), IL-6 (2.4 times), and MCP-1 (2 times), Fig 10. Elevated levels of IL-10 (3.2 times) were also detected, suggesting that the tissue had established a dual pro- and anti-inflammatory response cascade at this post-infection time. Furthermore, only slightly elevated expression of IFN-β (2 times) and associated molecules such as PKR (1.2 times) and STAT-1 (1.8 times) was observed (Fig 10). These results indicated that in our in vivo model, ZIKV neuroinfection induced a significant local immune response characterized by both pro-inflammatory and regulatory elements, associated with IL-10 and IFN-β.

Together, these findings highlight the complex and cell-type-specific nature of the immune response to ZIKV infection in the brain, were neurons emerged as key contributors to the inflammatory milieu, exhibiting robust expression of multiple immune markers, suggesting they may play a central role in orchestrating the local immune response. Overall, these results underscore the coordinated but differential activation of innate immune pathways among neurons, astrocytes, and microglia during ZIKV infection and point to neurons as potential primary drivers of neuroinflammation in this context.

## Discussion

After 10 dpi, neonatal mice inoculated i.c with a Colombian ZIKV strain, presented many infected neurons and microglia cells, with few astrocytes positive for the virus. This differs from other models that reported exclusive infection of either neurons [37] or glial cells [28]. Our infection model was non-lethal and non-microcephalic and resulted in a low number of viral RNA copies in the tissue. However, it induced notable changes in brain architecture, particularly in the cerebral vascular network. In infected animals, we observed an increase in both the number and diameter of vessels and capillaries in the cortex, possibly associated with an angiogenic process to meet the increased demand for oxygen and nutrient transport in the tissue. This vascular increase could also enhance the potential for ZIKV transport and transmission to other brain areas.

ZIKV infection can variably alter the vascularization of the brain and other susceptible tissues, depending on the developmental stage at which the infection occurs. Garcez et al, using intravenously infected pregnant Ifnar1^−^/^−^ mice at embryonic day (E) 12.5, observed a significant reduction in vascular density and branching, with an immature vascular pattern in both the brain and retina of E15.5 embryos [9]. In contrast, Shao et al. injected ZIKV directly into the cerebral ventricles of E14.5 embryos in immunocompetent C57BL/6 mice and reported a significant increase in vessel density and diameter in the cerebral cortex at birth (P0) [38].

These findings reinforce the teratogenic potential of ZIKV and highlight its stage-specific impact on brain development. Early gestational infections (E8–E10) predominantly affect vasculogenesis [39], likely through dysregulation of key inductive factors, resulting in impaired nutrient and oxygen delivery, reduced neural progenitor proliferation, cell cycle arrest, and apoptosis – hallmark features of ZIKV-associated microcephaly-. In contrast, later infections (E11–E15) primarily disrupt angiogenesis [40], and a similar pattern has been observed postnatally. In our model, which focused on the cerebral cortex, postnatal infection led to increased vessel number and dilation, accompanied by neuronal loss. Similarly, Figuereido et al. demonstrated that i.c. ZIKV inoculation at P0 in C57BL/6 mice impaired the postnatal development of the dentate gyrus (DG), leading to an increased number of blood vessels with reduced length and branching, along with diminished cellular proliferation and reduced DG volume (albeit without inducing microcephaly) [41]. These findings suggest that ZIKV, even in the neonatal period, can impair neurogenic niches such as the subgranular zone of the DG.

Importantly, insufficient and immature vasculature limits the availability of neurotrophic and metabolic support, thereby reducing neurogenesis and brain volume. Additionally, altered angiogenesis may contribute to neuropathology via immune-mediated mechanisms [42]. Although reactive gliosis has been consistently reported across models, our data uniquely indicates that neurons act as key responders and potential regulators of both inflammatory and resolution pathways in the nervous tissue. ZIKV neuroinfection predominantly induces a proinflammatory immune response, which may exacerbate neurological damage and contribute to clinical manifestations such as ataxia and paralysis.

Shao et al. further reported increased BBB permeability [38], a finding consistent with Jurado et al, who demonstrated that ZIKV infection of astrocytes leads to BBB disruption and CD8 ⁺ T cell infiltration into the brain parenchyma. Although CD8 ⁺ T cells reduced neuronal infection, they also induced paralysis, suggesting that both BBB breakdown and T cell–mediated neuropathology are key components of ZIKV-associated CNS injury [43]. BBB disruption has also been linked to a range of clinical manifestations, including myelitis [44], meningoencephalitis [45], lethal encephalitis [46], and acute flaccid paralysis, potentially associated with Guillain–Barré syndrome (GBS). This may result from demyelination, as reported in dorsal root ganglion models using Ifnar1^−^/^−^ mice [47], and/or the presence of anti-C1q antibodies in the serum of infected AG129 mice [48]. Plasma leakage into the parenchyma may also facilitate neuronal calcification via aggregation of serum proteins [49,50].

Additional factors, such as synaptic retraction (potentially reversible) and prolonged F-wave latencies in infected mice, point to the development of peripheral neuropathy between the sciatic notch and motor roots, without direct motor neuron loss. This mechanism could underline ZIKV-induced motor deficits [51].

In the neuroimmune context, these cells respond in a coordinated manner to eliminate the virus and any infected or affected cells, including neurons [52]. The elevated expression of MCP-1, TNF-α, and IL-6 suggests the establishment of an ambivalent response involving various cell populations such as astrocytes, microglia, and neurons. These cells produce cytokines, chemokines, and other factors that play crucial roles in both inflammatory processes and the resolution of nervous tissue injuries [53], as reflected by the immune cell infiltration and glial cell activation found.

Additionally, these cells participate in their own reparative processes due to their pleiotropic capacity to promote neuronal survival, proliferation, and the activation of glial cells [54]. This could explain the non-lethal nature of the infection, the low levels of viral replication, and the maintenance of the neuronal network. These observations were associated with the minimal changes noted in the CRMP-2 and MAP-2 expression and distribution, which are implicated in the growth and extension of the neuronal network [55]. Also, IL-10 was highly expressed, which has been involved in counteracting the pro-inflammatory effects of TNF-α and IL-6 while mitigating glutamate toxicity, factors responsible of neuropathogenic processes, including viral infections [56]. These findings suggest that ZIKV may induce a dual response potentially influenced by viral strain, initial viral load, and tissue maturity, which, in our model, appears to favor infection resolution.

Interestingly, the interferon-mediated response in the brain was relatively mild, likely reflecting the inherently low levels of IFN expression characteristic of Balb/c mice compared to other strains [37]. Nonetheless, a slight increase in IFN expression was observed at 10 days post-infection (dpi), supporting the hypothesis that the animals could mount a sufficient response to control the infection. However, ZIKV has been shown to evade antiviral responses through various mechanisms [37].

As a pleiotropic molecule, IFN acts synergistically with TNF-α and IL-10, indicating that the tissue’s response to ZIKV infection involves not only attempts to control viral replication but also the recruitment and activation of diverse immune cells at the infection site [37,57]. These observations emphasize that, despite certain limitations, our immunocompetent mouse model provides a valuable platform for studying the effects of ZIKV infection, offering insights into the intricate immune and cellular dynamics within tissue responses.

Of particular interest was the overexpression of STAT1 observed in both our *in vivo* and *in vitro* models. This finding suggests that during ZIKV infection, part of the IFN-mediated immune response might be activated as expected, despite the known viral evasion mechanisms. Interestingly, murine STAT1 and STAT2 demonstrate resistance to degradation by caspases and the proteasome, respectively, which are processes that ZIKV viral proteins, such as NS5, can typically promote [9,58,59] This supports the idea that ZIKV employs distinct immune evasion strategies in murine hosts. For example, ZIKV has been reported to modulate the activity of Dicer, a critical protein in the production of interference RNA (RNAi) [60] and utilize NS4B as an immune evasion strategy [61]. Additionally, STAT1 overexpression in our models may reflect the maturity state of the infected cells or animals, given STAT1’s known role in neurogenesis, proliferation, differentiation, and plasticity through the JAK/STAT signaling pathway [62]. These findings highlight the complex interplay between ZIKV immune evasion mechanisms and the host’s immune and developmental pathways.

Given the specific characteristics of our infection model, we chose to use one-day-old mice of the same strain to evaluate the response patterns of three key cell types involved in ZIKV pathogenesis, which are also important for establishing the immune response in nervous tissue. Before infection, cultures achieved a high degree of purity (95−98%), suggesting that the observed responses were predominantly orchestrated by a single cell type. Additionally, the *in vitro* differentiation process yielded a high proportion of mature cells. Cultured cortical neurons displayed a differentiated phenotype (Sox2-negative) with characteristic neurite and axon extension over time, as evidenced by CRMP-2 expression, a key factor involved in axon guidance, synapse maturation, and neuronal migration [55]. Additionally in neurons, the expression of MAP2 and CRMP-2 was evaluated. The co-expression of MAP2 and CRMP-2 highlights the neurons’ structural integrity and functional maturity. Specifically, MAP2 expression ensures the stability and maintenance of dendritic architecture, while CRMP-2 expression indicates that cultured neurons are actively involved in processes related to axonal growth, guidance, and proper neural circuit formation [63–65]. Astrocyte cultures also exhibited high purity, with 98% expressing GS, a key enzyme in astrocyte energy metabolism [66], and 95% expressing GFAP, a marker of astrocyte differentiation [67]. Achieving a highly advanced in vitro differentiation of these cells allowed us to study ZIKV infection dynamics in postnatal nervous tissue cells.

We found cortical neurons did not survive high viral doses, so we evaluated only the least lethal viral multiplicities of infection (MOIs 0.1 and 0.5) at 48 hpi. We observed slight changes in the neuritic network, as previously reported by Shao et al., who evaluated the infection effect on microtubule-associated proteins without observing changes in the neuronal network of infected brains [38]. Additionally, we observed high intra- and extracellular virus production, confirming ZIKV’s ability to infect differentiated neurons, as reported in adult mouse models or in human tissue section [41]. However, viral RNA copies did not decrease over time, suggesting an inability of the antiviral system to control viral replication. This has been previously proposed in other models, where an inhibition of molecules associated with the signaling pathway activated by IFN occurred due to the action of non-structural viral proteins such as NS3 [58,68,69], evading the antiviral signaling process that involved PKR and STAT-1, which explain their low expression levels in the different cell types. However, PKR regulation during ZIKV infection is also associated with the development of tauopathies [37], making it a molecule of interest that might help to explain, for example, the neurological impairment in non-microcephalic children after *in utero* infection.

On the other hand, our differentiated astrocyte and microglia cultures were susceptible to ZIKV without affecting their survival, although viral RNA copies (intra- and extracellular) were low. The recorded glial cell response was enthralling, showing a low viral replication rate and a pro-inflammatory pattern associated with IL-6, TNF-α, and MCP-1, suggesting a positive feedback loop between neurons and glial cells that amplified the pro-inflammatory environment in the brain, as proposed by Manet et al. [37].

Regarding astrocytes, it appears that the virus can exquisitely modulate several mechanisms that activate and regulate astrocytes innate immune response, associated with the low infection rate and low viral production, suggesting a role as viral reservoirs, which can explain the potential use as therapeutic strategy for glioblastoma brain tumors [70,71]. This dissimilar response pattern between neurons and glial cells indicates that despite supporting viral replication, the innate immune response established by each cell type is independent of viral load and replicative capacity. This suggests that glial cells have a stronger antiviral immune inhibition response exerted by type I interferons than neurons. It is noteworthy that we use a ZIKV strain that circulated during the American outbreak of 2016, which showed different clinical severity and sequelae frequency regarding Asiatic or African variants and could explain the diverse responses to the virus according to the strain used in the experiments, which determine both viral tropism and cell type response.

Variations in ZIKV tropism and fitness have been attributed to mutations accumulated in its genome during replication across different tissues and hosts [72]. Among them, the H401Y mutation in the E protein has been shown to increase virulence, neurotropism, and lethality in mouse models, and has been identified in isolates from mosquitoes and humans in Asia and the Americas, where neurological complications associated with ZIKV were more frequent [73]. Moreover, ZIKV has been shown to evolve within a single host. A recent study detected brain-specific mutations—absent in other tissues—associated with non-structural proteins (NS2A, NS1, and NS4B) linked to neuroinfection and neuropathogenesis [74]. These findings support the quasispecies model, in which each viral inoculum represents a diverse population of genomes that act cooperatively or competitively to enhance viral adaptation [75,76] This diversity allows ZIKV to maintain broad tropism without losing vector infectivity and facilitates adaptation through mutations that promote efficient cell entry, replication, and modulation of the antiviral response [74].

Both *in vitro* and *in vivo* models confirm that differentiated nervous tissue cells are susceptible to ZIKV, demonstrating the virus’s potential to infect and primarily alter neurons even in advanced developmental stages. Additionally, taken together, the in vivo and in vitro experiments suggest that the immune response may be bivalent, involving glial activation and the expression of both pro- and anti-inflammatory molecules, mainly in microglia. In vivo, this response may contribute to resolving the infection and preserving the neuronal network, whereas in vitro, the isolated response appears insufficient, coinciding with neuronal death and damage.

Further *in vivo* and *in vitro* studies are required to understand the behavior of ZIKV circulating in Colombia, where 106,552 cases of Zika fever were reported between 2015 and 2016, 18.5% of which reported in pregnant women [77], resulting in 710 cases of microcephaly in children (4.87 cases per 10,000 live births) [78]. This placed Colombia as the second most affected country by the ZIKV epidemic, after Brazil.

As mentioned above, exposure to ZIKV during different stages of pregnancy leads to two distinct outcomes. The most extensively studied and widely recognized is microcephaly, along with other severe tissue malformations, which clearly demonstrate the virus’s damaging effects on nervous tissue. The second, less understood outcome involves subtle neurological alterations that become apparent only during the later stages of postnatal development, in children who do not exhibit overt morphological abnormalities, at least not detectable with current diagnostic tools.

To date, the few studies that report long-term postnatal neurological sequelae have been conducted in animal models. In mice, spontaneous seizures during the juvenile period, learning impairments, and increased susceptibility to chemically induced seizures in adulthood have been observed [24]. Similarly, chronic behavioral, motor, and cognitive abnormalities—such as heightened emotional reactivity, reduced social interaction, impaired balance, and deficits in visual recognition memory—have been documented in nonhuman primates [79] further highlighting the need for continued investigation into the neurodevelopmental consequences of ZIKV infection. Therefore, animal models such as the one used in our study—based on the infection of one-day-old mice, which developmentally correspond to the third trimester of human gestation [23,24] are essential to investigate how ZIKV may induce cognitive and behavioral alterations through the disruption of neuronal networks. This developmental window is characterized by active cortical maturation, neuronal and glial migration, and the establishment of neuronal circuits that are critical for motor, sensory, and cognitive functions [80,81]. Notably, these changes occur without any detectable reduction in skull size or other signs typically measurable during routine pediatric evaluations.

These findings, along with our results, highlight the need to continue studying ZIKV’s effects and its interactions with other circulating flavivirus like yellow fever virus, and dengue virus, as immunity to these viruses could influence infection rates and placental, fetal, and postnatal nervous system infections.

## Limitations

This study confirms the ability of ZIKV to infect nervous tissue cells during postnatal developmental stages in immunocompetent mice, demonstrating that ZIKV pathogenic and teratogenic effects extend beyond microcephaly. While our findings underscore the utility of murine models, we also acknowledge several limitations and areas for further investigation:

- Applicability to Human Pathology: As with any animal model, findings in mice may not fully capture the cellular and molecular factors involved in ZIKV infection and postnatal damage in humans. However, our study did not evaluate learning behavior, epileptiform activity, or auditory and visual sensory deficits, which could reflect non-microcephalic neurological alterations that may manifest after 10 days post-infection
- Strain Variability: The results were obtained using a strain of ZIKV propagated in C6/36 cells. Future studies should compare these findings with other ZIKV strains of diverse geographical origins to understand strain-specific differences.
- Innate Immune Response: The divergent mechanisms of ZIKV in the murine model necessitate further evaluation of the expression and function of additional players in the innate immune response, such as neurons, astrocytes, and microglia, including their interactions and mutual regulation.
- IFN Family and Associated Pathways: It is crucial to assess the expression and modulation of other IFN family members, along with associated proteins and genes activated by these pathways, during postnatal infection. Comparing these responses with cells at earlier developmental stages (embryonic) and fully differentiated cells (e.g., at 10 days of age) would provide insight into developmental stage-specific effects.
- Impact on Differentiation and Function: Evaluating how ZIKV affects processes related to the differentiation and function of neuronal and astrocytic populations and subpopulations could reveal additional mechanisms of neural disruption.
- Endothelium and Blood-Brain Barrier: The impact of ZIKV infection on endothelial cells and the integrity of the blood-brain barrier needs further exploration to understand how these structures contribute to and are affected by infection.

## Conclusions

*In vivo*, ZIKV infection was not lethal but resulted in a substantial number of infected neurons and microglia. The low viral replication and cytokine profiles suggest a somewhat controlled inflammatory response that may prevent severe damage to the neuronal network, explaining the survival of the mice. We hypothesize that *in vivo*, non-microcephalic alterations induced by ZIKV may be associated, among other factors, with impaired angiogenesis and increased vascular permeability. These changes could contribute to tissue damage through mechanisms such as immune cell infiltration and the release of inflammatory mediators that trigger immune activation. Further studies are needed to better understand the postnatal effects of ZIKV infection and the full spectrum of its neurological manifestations.

*In vitro*, it was evident that astrocytes and microglia responded to infection in different ways, helping to establish a positive feedback loop that appears to amplify the pro-inflammatory environment. In both models, neurons were the primary cell type infected and affected by ZIKV. Neuronal infection is associated with pronounced inflammatory responses and substantial cell death, particularly at higher viral loads. The persistent activation of pro-inflammatory pathways and the evasion of antiviral responses by ZIKV underscore the severity of its impact on neuronal health. The observed alterations in neuronal network proteins suggest disruptions in neuronal connectivity and function, which may contribute to neurological and behavioral alterations. Further research is essential to fully understand these effects and develop targeted strategies to mitigate the impact of ZIKV on the nervous system even in subclinical infections.

## Supporting information

S1 Fig Viral detection in mouse brain lysates using A549 cells. Cleared supernatants from infected mouse brain lysates were collected at 10 dpi and applied to A549 cells, which were incubated for 72 h. Then, cells were fixed, and viral antigens were detected by immunoperoxidase assay. Non-infected and mock-infected cells served as negative controls while ZIKV-infected cells (MOI 0.1) were included as a positive control. Representative images from two independent experiments performed in triplicate are shown. Scale bars: 100 µm for non-infected control and non-infected/mock brain lysates; 50 µm for all other conditions. Raw infection counts can be found at https://doi.org/10.7910/DVN/GT2BTZ, Harvard Dataverse.
(TIF)

S2 Fig Neuronal damage in ZIKV infected brains. Brain sections were analyzed to quantify the number of cells with ND, including pyknosis, karyorrhexis, karyolysis, and apoptosis using Fiji/ImageJ. The number of ND cells per condition as well as the percentage equivalent are shown. Statistical significance was assessed using the Mann–Whitney test, ****p < 0.0001.
(TIF)

S3 Fig Microglial cell isolation and morphology in culture. Mixed glial cultures were maintained for 15–18 days before microglial cells were harvested. A) Panoramic view of the mixed glial cultures at day 18 (20X magnification). B) Higher magnification view (40X) of microglial cells in mixed cultures. (C) Upon reaching confluence, microglial cells were purified by constant shaking for 2 h, collected, and reseeded. Representative images from three independent cultures are shown. Scale bars: A and C, 200 µm; B, 100 µm.
(TIF)

S4 Fig Viral detection in primary cell culture supernatants using A549 cells. Cleared supernatants from ZIKV (MOIs 0.01–3) infected neurons, astrocytes and microglia cell cultures were collected at 48 hpi and applied to A549 cells, which were incubated for 72 h. Then, cells were fixed, and viral antigens were detected by immunoperoxidase assay. Representative images from two independent experiments performed in triplicate are shown. Scale bars: 100 µm or 50 µm. Raw infection counts can be found at https://doi.org/10.7910/DVN/GT2BTZ, Harvard Dataverse.
(TIF)
