# Supplementary figures and images for: In vivo and in vitro susceptibility and inflammatory response of postnatal mouse cortical neurons and glial cells to zika virus infection
Source: PLoS One. 2025 Dec 31;20(12):e0339900. doi: 10.1371/journal.pone.0339900 (PMC12755826; doi:10.1371/journal.pone.0339900)

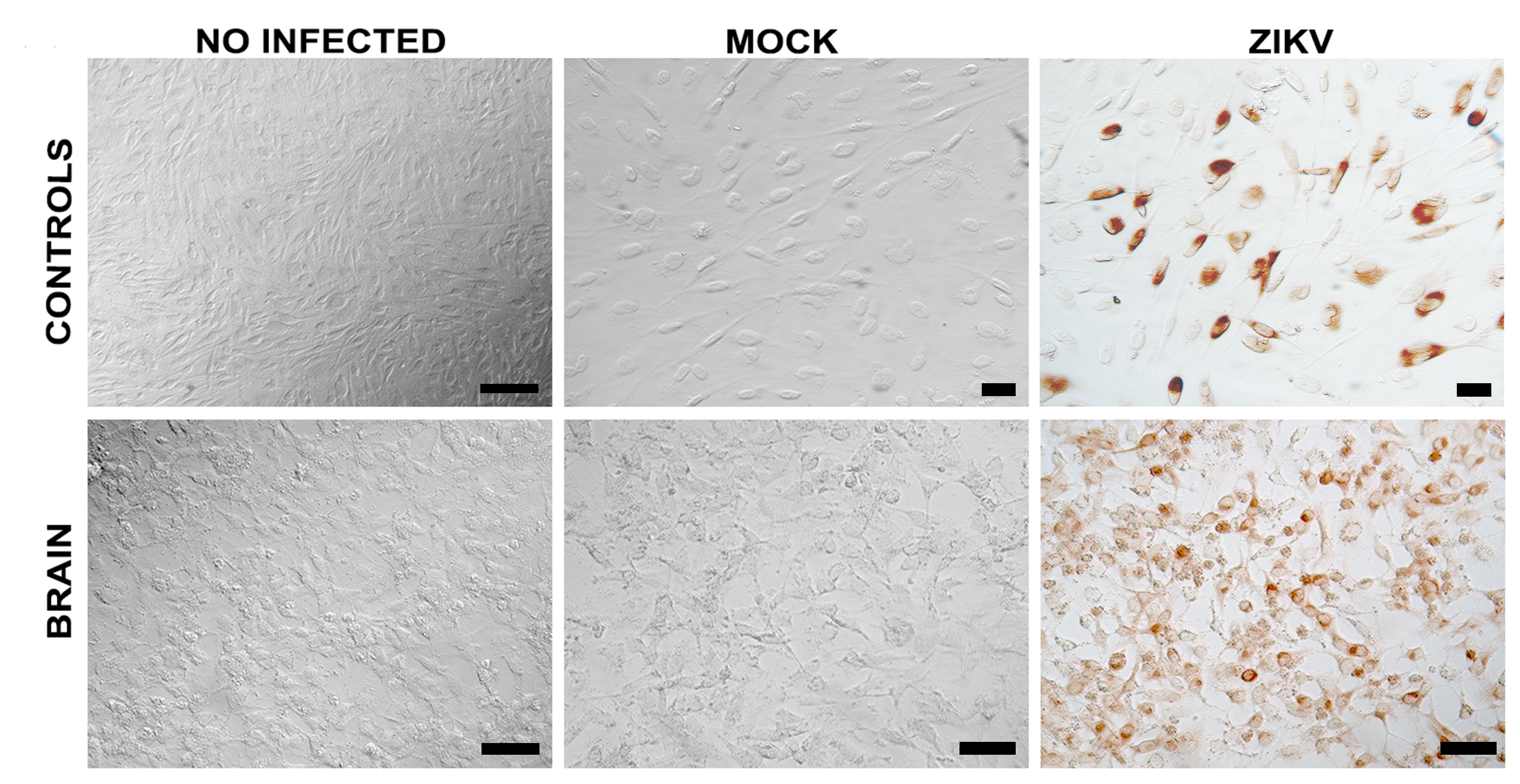

Supplement: S1 Fig — Cleared supernatants from infected mouse brain lysates were collected at 10 dpi and applied to A549 cells, which were incubated for 72 h. Then, cells were fixed, and viral antigens were detected by immunoperoxidase assay. Non-infected and mock-infected cells served as negative controls while ZIKV-infected cells (MOI 0.1) were included as a positive control. Representative images from two independent experiments performed in triplicate are shown. Scale bars: 100 µm for non-infected control and non-infected/mock brain lysates; 50 µm for all other conditions. Raw infection counts can be found at https://doi.org/10.7910/DVN/GT2BTZ, Harvard Dataverse. (TIF) [file pone.0339900.s001.tif]

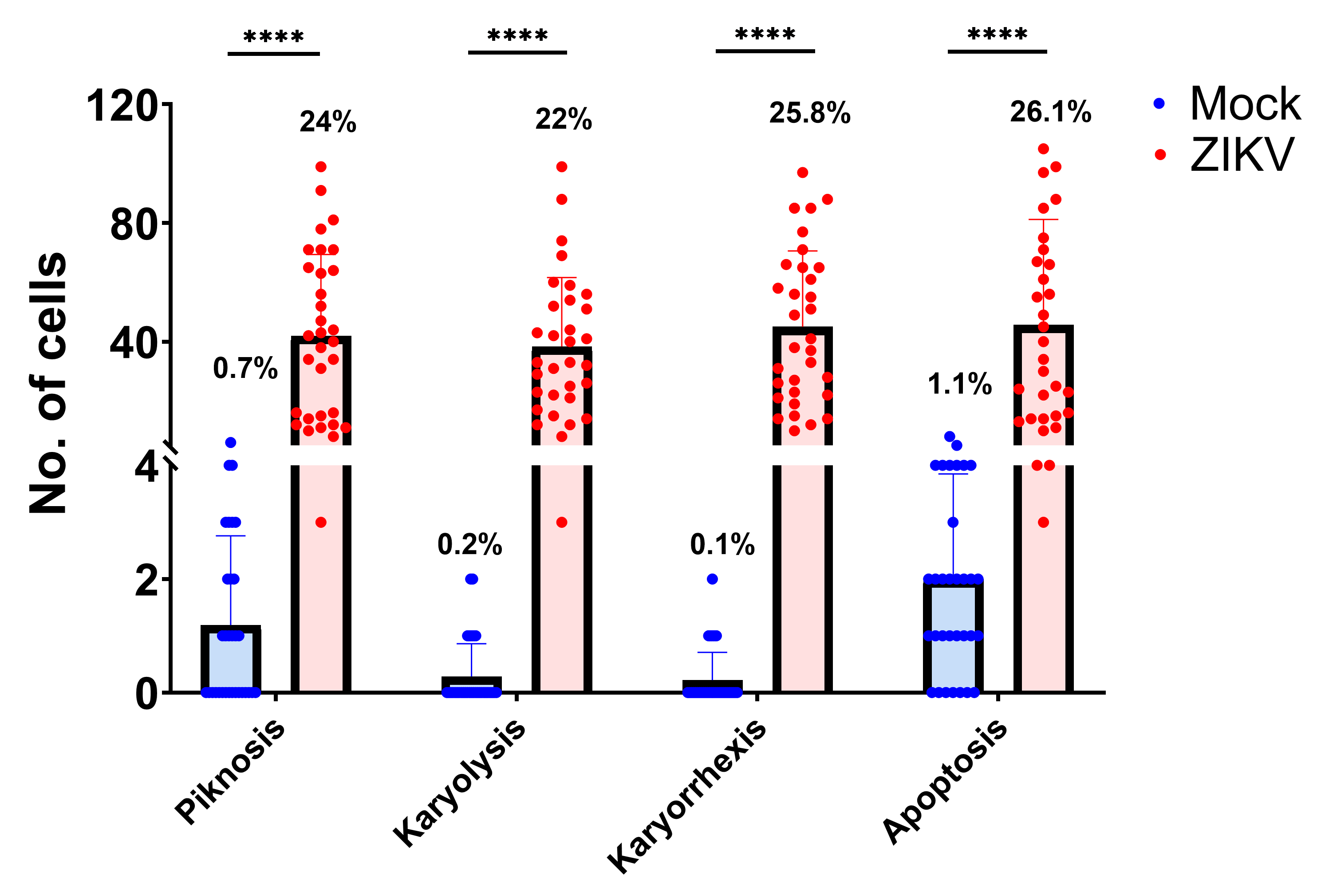

Supplement: S2 Fig — Brain sections were analyzed to quantify the number of cells with ND, including pyknosis, karyorrhexis, karyolysis, and apoptosis using Fiji/ImageJ. The number of ND cells per condition as well as the percentage equivalent are shown. Statistical significance was assessed using the Mann–Whitney test, ****p < 0.0001. (TIF) [file pone.0339900.s002.tif]

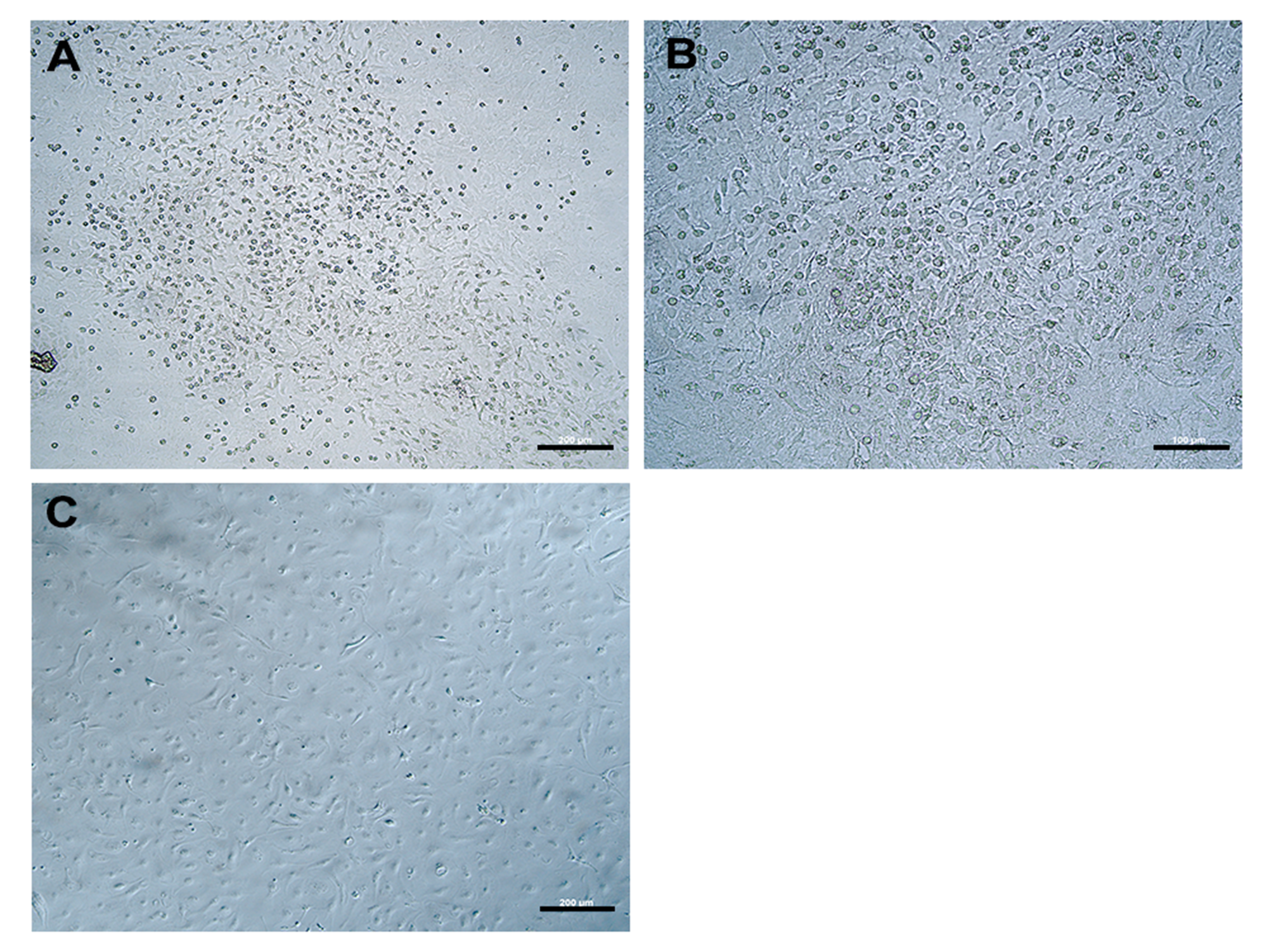

Supplement: S3 Fig — Mixed glial cultures were maintained for 15–18 days before microglial cells were harvested. A) Panoramic view of the mixed glial cultures at day 18 (20X magnification). B) Higher magnification view (40X) of microglial cells in mixed cultures. (C) Upon reaching confluence, microglial cells were purified by constant shaking for 2 h, collected, and reseeded. Representative images from three independent cultures are shown. Scale bars: A and C, 200 µm; B, 100 µm. (TIF) [file pone.0339900.s003.tif]

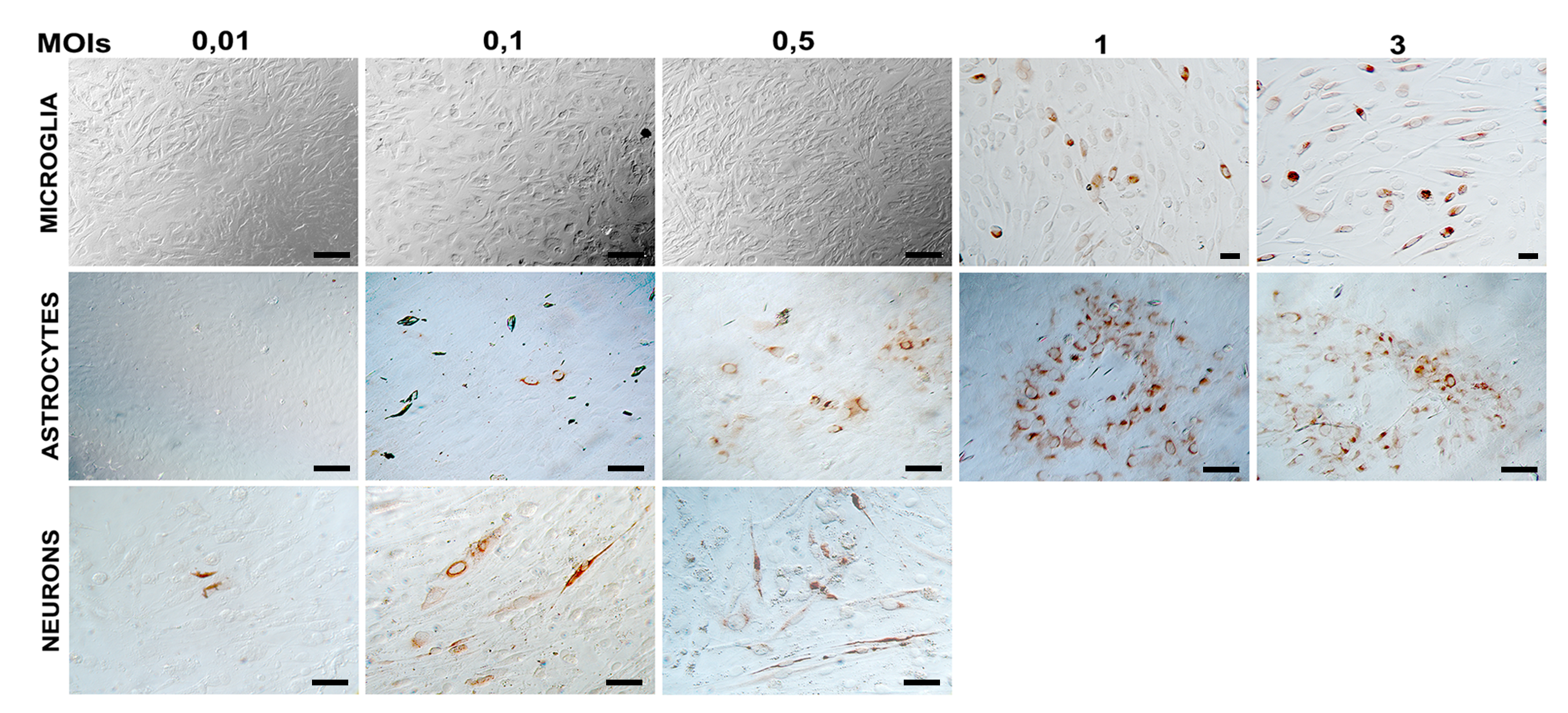

Supplement: S4 Fig — Cleared supernatants from ZIKV (MOIs 0.01–3) infected neurons, astrocytes and microglia cell cultures were collected at 48 hpi and applied to A549 cells, which were incubated for 72 h. Then, cells were fixed, and viral antigens were detected by immunoperoxidase assay. Representative images from two independent experiments performed in triplicate are shown. Scale bars: 100 µm or 50 µm. Raw infection counts can be found at https://doi.org/10.7910/DVN/GT2BTZ, Harvard Dataverse. (TIF) [file pone.0339900.s004.tif]
